# Supplementary material for: Diagnose-Specific Antibiotic Prescribing Patterns at Otorhinolaryngology Inpatient Departments of Two Private Sector Healthcare Facilities in Central India: A Five-Year Observational Study
Source: Int J Environ Res Public Health. 2019 Oct 23;16(21):4074. doi: 10.3390/ijerph16214074 (PMC6862163; doi:10.3390/ijerph16214074)
Supplement: Supplementary file 1 [file ijerph-16-04074-s001.pdf]

Table S1. Classification of the ENT-inpatients in Surgical diagnoses sub-groups and Non-surgical diagnoses sub-groups

Group A: Surgical diagnoses			Group B: Non-surgical diagnoses		
**Subgroup A1**	**Subgroup A2**	**Subgroup A3**	**Subgroup B1**	**Subgroup B2**	**Subgroup B3**
Contaminated/dirty surgery	*Clean-contaminated surgery*	*Clean surgery*	*Clinical infection of bacterial, fungal or parasitic origin*	*Clinical infection of viral origin*	*Non-infectious diseases*
Abscess<br>Trauma<br>Infected cysts<br>Fractures<br>Open wounds<br>Dental root & gum abscess<br>Peritonsillar abscess<br>Cellulitis in face<br>Septal hematoma<br>Chronic rhinosinusitis<br>Ludwig´s Angina<br>Osteomyelitis<br>Dacryocystitis	Radical resection head and neck cancer, i.e., buccal mucosa, tongue, tonsils, nasal, parotid, skin<br>Deviated nasal septum<br>Nasolabial cyst<br>Tympanoplasty<br>Eagle´s syndrome<br>Embedded tooth<br>Bronchial cyst<br>Epidermoid cyst<br>Oral and cervical ranula<br>Mucous retention cyst<br>Septal perforation<br>Lipoma, haemangioma & angiofibroma<br>Tm joint ankyloses<br>Trismus<br>Epulis	Biopsies, i.e., oral ulcers, leukoplakia, larynx, neck secondary, biopsy of cancers<br>Tonsillectomy<br>Foreign bodies<br>Thyroid surgery, i.e., goiter, nodule, adenoma<br>Earlobe reconstruction<br>Aural polyp<br>Adenoid hypertrophy<br>Micro-laryngeal surgery<br>Non-infectious cysts<br>Parotid fistula<br>Nasal endoscopy, i.e., polyps, deviated nasal septum<br>Dysphagia<br>Skin-surgery<br>Thyroglossal cyst<br>Tongue tie<br>Oesophagus dilatation for achalasia cardia	Tuberculosis<br>Acute mastoiditis<br>Sialadenitis<br>Leprosy<br>Sepsis<br>Rhinoscleroma<br>Meningitis<br>Preseptal cellulitis<br>Eye pus UNS<br>Perichondritis<br>Acute otitis media<br>Pansinusitis<br>Acute rhinosinusitis<br>Acute sinusitis of bacterial or fungal origin<br>Acute tonsillitis<br>Acute supraglottitis<br>Acute epiglottitis<br>Pneumonia<br>Glossitis<br>Otomycosis<br>Otomycosis<br>Nasal myiasis<br>Discharge ear NES	Throat pain NES<br>Acute pharyngitis<br>Upper respiratory tract infection UNS<br>Viral fever<br>Varicella-zoster virus diseases<br>Herpes zoster virus diseases<br>Throat infection NES<br>Acute parotitis<br>Acute laryngitis<br>Chronic obstructive pulmonary disease	Neck or head swelling<br>Epistaxis<br>Anemia<br>Hypo/hyperthyroidism<br>Neurological diseases<br>Impacted cerumen<br>Meniere's disease<br>Presbyacusis<br>Hearing loss NES<br>Otalgia NES<br>Vasomotor rhinitis<br>Pneumothorax<br>Allergic rhinitis<br>Dyspepsia<br>Skin disorders<br>Dysphagia due to iron deficiency<br>Vertigo<br>Fever NES<br>Pain NES<br>Superficial Injury<br>Tm joint dislocation<br>Allergic reactions<br>Mouth Bleeding NES<br>Adhesive otitis media

Abbreviations: A1 = contaminated/dirty surgery; A2 = clean-contaminated surgery; A3 = clean surgery; ENT = ear, nose and throat (otorhinolaryngology); NES = Not elsewhere specified; UNS = unspecified. The classification is based on Scottish Intercollegiate Guidelines Network (2014), Bratzler D, Dellinger E, Olsen K et al. Clinical practice guidelines for antimicrobial prophylaxis in surgery. Am J Health Syst Pharm 2013; 70: 195-283, and Xavier Ottoline A, Tomita S. Antibiotic prophylaxis in otolaryngologic surgery. Int Arch Otorhinolaryngol 2013; 17: 85-91.
